# Beyond-hot-spot absorption enhancement on top of terahertz nanotrenches

**DOI:** 10.1515/nanoph-2022-0214

**Published:** 2022-05-25

**Authors:** Jeeyoon Jeong, Dai-Sik Kim, Hyeong-Ryeol Park

**Affiliations:** Department of Physics and Institute of Quantum Convergence Technology, Kangwon National University, Gangwon 24341, Republic of Korea; Department of Physics and Astronomy, Seoul National University, Seoul 08826, Republic of Korea; Department of Physics, Ulsan National Institute of Science and Technology (UNIST), Ulsan 44919, Republic of Korea

**Keywords:** absorption, field enhancement, hot spots, nanogaps, terahertz

## Abstract

Metallic nanogaps are being widely used for sensing applications, owing to their ability to confine and enhance electromagnetic field within the hot spots. Since the enhanced field does not confine itself perfectly within the gap, however, fringe fields well away from the gap are of potential use as well in real systems. Here, we extend the concept of near field absorption enhancement by quantitatively analyzing terahertz absorption behavior of water molecules outside the hot spots of sub-20 nm-wide, ∼100 μm-long nanotrenches. Contrary to point-gaps which show negligible field enhancement at distances larger than the gap width, our extended nanogap act as a line source, incorporating significant amount of absorption enhancement at much longer distances. We observe absorption enhancement factors of up to 3600 on top of a 5 nm-wide gap, and still well over 300 at 15 nm away. The finding is well supported by theoretical analyses including modal expansion calculations, Kirchhoff integral formalism and antenna theory. Our results provide means to quantitatively analyze light-matter interactions beyond the hot spot picture and enable application of nanogaps for sensitive surface analyses of various material systems.

## 1 Introduction

Local field enhancement at hot spots of metallic nanostructures can enhance various types of light-matter interactions including Raman scattering [1–4], molecular absorption [5–7], and harmonic generation [8–10], etc. To find out the mechanisms of such processes one needs to exactly quantify the field enhancement factors, which is difficult to obtain experimentally and is usually aided by electromagnetic simulations. Background-free metallic nanogap structures such as nanotrenches are unique in such context [11–13], as the near field enhancement at the hot spot can be unambiguously linked to observed far-field transmission via Kirchhoff integral formalism [14]. Such structures have been extensively investigated in various configurations and have been found especially useful in quantifying absorption enhancement [15–17], optical tunneling [18–20] and rectification [21, 22].

Such analyses, however, mostly treat the light-matter interaction as occurring within the hot spot, or within the gap volume, neglecting the contribution from fringe field outside the gap. This approach is valid only when materials of interest are solely contained inside the hot spots of metallic nanostructures, while many realistic nanogap-material hybrid structures inevitably contain some portion of target material outside the gap. In most cases, contribution from outside the gap is not significant as fringe fields decay quickly away from the gap; for instance, surface plasmon modes decay exponentially in out-of-plane direction. However, when the gap size is as small as length scale of the target material itself, as in many bio-applications and surface analyses, the material can at best lie on top of the nanogaps and the fringe field is the only source for the enhancement of light–matter interaction [23–27]. Therefore, means to maximize the fringe fields and to quantitatively analyze the light–matter interaction is essential for application of metallic nanostructures in many realistic systems.

**Figure 1:**
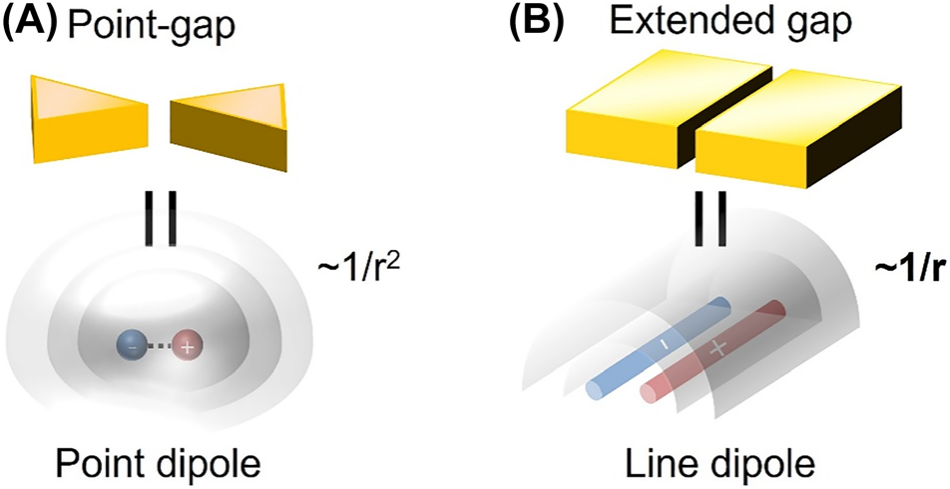
Schematic description of field distribution outside a point-gap and an extended gap. (A) In a point gap, energy conservation dictates that Poynting vector of the spherical scattering wave from a point-gap falls off as 1/*r* ^2^. (B) Scattering from an extended gap, on the other hand, incorporates ∼1/*r* dependence (cylindrical wave). Therefore, a long, narrow nanotrench structure can utilize much larger fringe fields for beyond-hot-spot absorption enhancement.

In this work, we utilize 5∼20 nm-wide, ∼100 μm-long nanotrenches to boost molecular absorption at distances well away from the gap and to quantitatively understand the enhancement mechanism. We focus on the fact that scattering from nanogaps with extreme sub-wavelength widths and high aspect ratio can be approximated as a cylindrical wave, in contrary to the case of more commonly used point-gaps where the scattering is frequently modeled as a point dipole source (Figure 1). Therefore, the high aspect ratio nanogaps are expected to incorporate fringe fields that decay much slower, such that light–matter interaction can be sufficiently enhanced at distances far away from the gap (i.e., several times the gap width). With quantitative understanding on such phenomena, our structure may enable new nanophotonic applications that extend beyond the familiar picture of hot spots.

**Figure 2:**
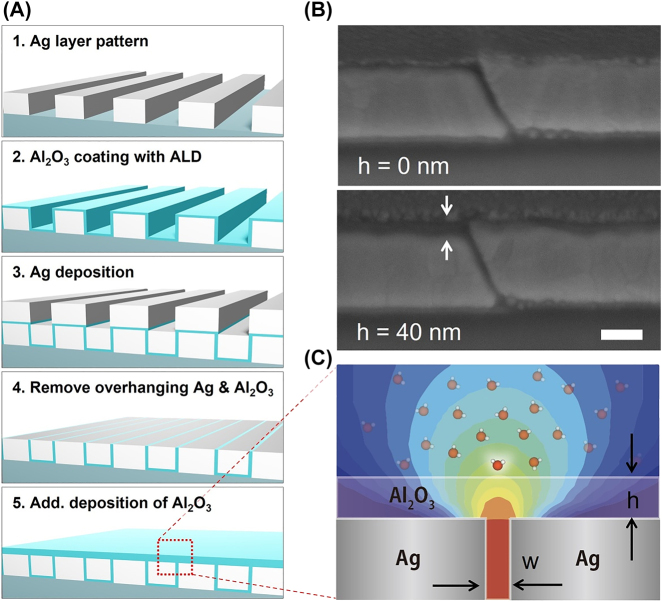
Nanometric control of distance between the nanotrenches and water molecules. (A) Fabrication process for the nanotrenches. On an Ag layer patterned by photolithography and lift-off, alumina is conformally coated on the whole structure with atomic layer deposition (ALD) technique (step 1, 2). Subsequent deposition of additional Ag fills the void, and Ag–alumina–Ag gaps are formed at the sidewalls with gap widths equal to the thickness of deposited alumina (step 3). Mechanical exfoliation and subsequent angled dry etching cleans up the surface (step 4), and distance from the gap is controlled with additional deposition of alumina with ALD (step 5). (B) Cross-sectional scanning electron microscope image of a 10 nm wide gap sample before (top) and after (bottom) deposition of 40 nm alumina on top. (C) Schematic figure showing absorption enhancement for water molecules outside the hot spots of the gap.

In our experiment, a nanotrench-water assembly comprising 4 layers – a glass substrate, metallic nanotrenches, a 15 μm-thick water layer, and a glass cover – is prepared, and terahertz waves transmit through the assembly from the substrate side. The metallic nanotrenches are in the form of an array of 20 × 80 μm rectangular rings on 200 nm thick silver films, with varying gap widths of 5, 10, and 20 nm. Distance between each ring is 20 μm, such that periods are 40 μm and 100 μm in the two directions. The nanotrenches are fabricated via a spacer-based method commonly referred to as atomic layer lithography [28], details of which are as follows (Figure 2A). First, 200 nm thick Ag layers with an array of 20 × 80 μm rectangular holes are patterned on top of Pyrex glass substrates using standard photolithography (AZ5214E, Microchem) and lift-off technique. Through subsequent atomic layer deposition (ALD) process, amorphous aluminum oxide (alumina) layer of 5, 10, and 20 nm thicknesses conformally covers the whole structure, creating dielectric membranes on top and on sidewalls of the patterned Ag layers. Then 200 nm Ag is deposited over the whole structure, filling the holes, and creating Ag–alumina–Ag gaps on the sidewalls of the Ag pattern, where the gap width is defined by the thickness of the alumina layer. Excess metal and alumina on top of the first Ag layers is removed by exfoliation with scotch tape and Argon ion milling at an oblique angle (∼80°), thereby revealing the vertically aligned gap. Since the gap is filled with alumina, no water molecules enter the gap and only the fringe fields outside the gaps contribute to the interaction with the absorbing liquid layer on top. Afterwards, additional deposition of alumina with ALD nanometrically controls the gap-water distance (Figure 2B).

The water reservoir on top of the nanotrenches is realized by a cover layer with a spacer, fabricated from spin-coated polydimethylsiloxane (PDMS) on a sapphire substrate. Liquid-phase PDMS is first prepared by proper mixing and degassing of base and curing agent (10:1 ratio, Sylgard 184 silicone elastomer kit). The PDMS is drop-cast on a sapphire substrate and then spin-coated at 1500 rpm for 30 s to form a 15 μm-thick film. Baking the PDMS-coated substrate at 120 °C for 15 min and cutting out unwanted parts lead to a 15 μm-deep room for liquid water.

Transmission spectrum of the sample is measured with a home-made terahertz-time domain spectroscopy setup. Due to extremely high permittivity of metal at low frequencies, terahertz radiation can barely pass through the metal film and transmits only through the gap, which greatly simplifies the analyses. For generation of terahertz pulses (0.01–3 THz), a 150 fs pump pulse of 80 MHz repetition rate and 800 nm center wavelength is incident on a 150 V-biased gallium arsenide photoconductive antenna. The terahertz wave is focused onto a spot approximately 3 mm in diameter and passes through a 2 mm × 2 mm square aperture before transmitting though the sample. The small aperture ensures that the incident beam can be approximated as a plane wave, and that possible experimental artifacts from oblique incident angle can be ignored. The transmitted beam is collected with off-axis parabolic mirrors and detected by electro-optic signal from a zinc telluride crystal.

When the terahertz radiation is incident on the nanotrench-water assembly, terahertz waves funnel through the gap and then pass through the absorbing water layer. The electromagnetic fields on top of a nanotrench will quickly fade away as a function of distance from the gap exit, leading to a different degree of electromagnetic absorption enhancement for water molecules at different positions (Figure 2C). Then, as the distance between the gap and water is nanometrically increased via additional ALD of alumina on top of the gap where fringe field exists, total absorption by the water layer will decrease, which will be observed as an increase in overall transmission. Relating the known field profile from the gap and the changes in absorption will enable quantitative analysis on the near-field absorption phenomena. It should be noted that we deposited the additional alumina layer on a single nanotrench sample and repeated the experiment multiple times rather than preparing multiple samples with different gap-water distances; this was possible due to excellent properties of our cover layer for the water reservoir (see Supporting Information). It is also worth noting that alumina minimizes the reflective effect by index-matching onto the water layer, as they have almost identical refractive indices in terahertz frequencies (∼2.3).

## 2 Results and discussions

**Figure 3:**
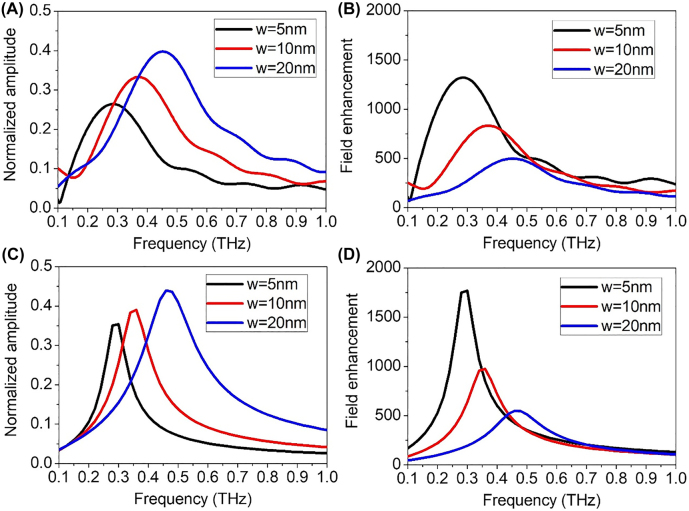
Transmitted electric field amplitude spectra of nanotrenches with various gap widths. (A) Measured transmittance spectra for 5, 10, and 20 nm-wide nanotrench samples. Each spectrum is normalized with respect to transmittance of bare glass substrate. (B) Field enhancement factors at the gap exit retrieved from experimental data using Kirchhoff integral formalism. (C) Calculated values for normalized transmittance, and (D) corresponding field enhancement factors.

Conventional terahertz time domain spectroscopy is used to characterize the transmittance of the samples. Terahertz frequency is well suited for the experiment because direct transmission through metallic film is negligible in this frequency range, enabling exact quantification of field enhancement factors without having to account for a background signal. This is well demonstrated in Figure 3A where normalized transmittances of the nanotrench samples reach up to 40% while direct transmittance through a bare 200 nm-thick silver film is less than 0.3% (not shown). Also, we make complementary use of Kirchhoff integral formalism and modal expansion calculation to determine the field amplitude over the whole region [12, 29], [30], [31]. The former relates the experimentally observed far field transmittance to the near field enhancement at the gap exit, while the latter gives an analytical solution to the field profile over the whole space (see Supporting Information for details). Note that the extreme width-to-length aspect ratio of the nanotrench (∼1:16,000) hinders usage of common electromagnetic simulation tools such as finite difference time domain method. Figure 3B shows the field enhancements of the nanogap samples deduced from the transmittance data using the Kirchhoff integral formalism. As direct transmission through metal film can be ignored, all transmission must come from the gap and *E*_enh_ = *t*/*β*, where *E*_enh_ is near field enhancement at the gap, *t* is normalized far field transmittance (in terms of electric field amplitude, not intensity), and *β* is the areal coverage ratio of the gap over the metal, *A*_gap_∕*A*_metal_, where *A* denotes the area of gap or metal covering the sample. Figure 3C and D show the corresponding results from modal expansion method, validating the reliability of the calculation via well-matched spectra.

**Figure 4:**
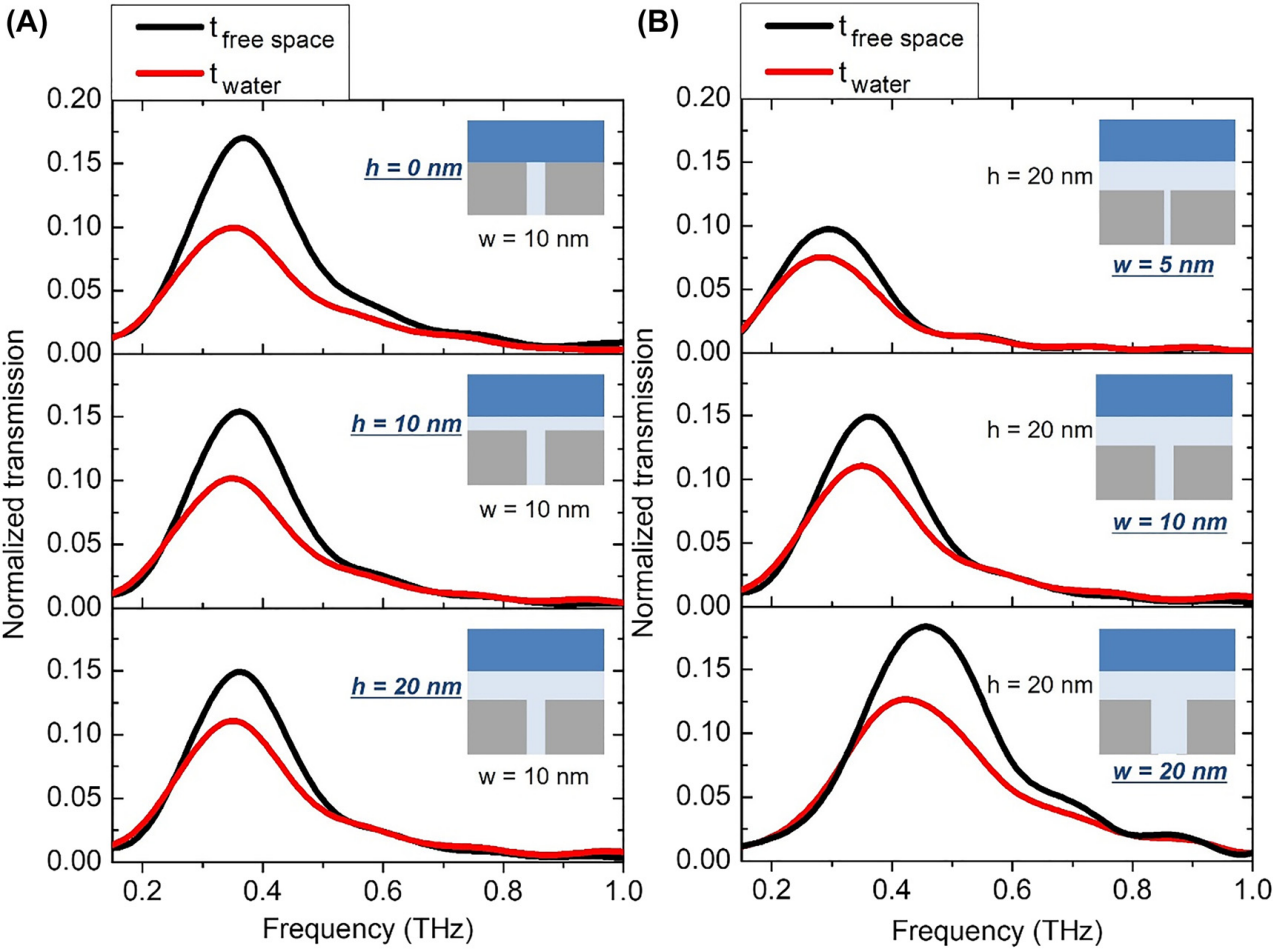
Normalized transmission spectra of (A) 10 nm-wide gap sample with varying gap-water distances of 0, 10, and 20 nm, and (B) 5, 10, and 20 nm-wide gap samples with a fixed gap-water distance of 20 nm. Here the normalized values are in terms of intensity rather than field amplitude, for more direct conversion into absorption in next steps.

As water layer is placed on top of the nanotrenches, the transmission decreases due to the enhanced terahertz absorption from the water layer, the amount of which differs depending on the gap width and the gap-water distance. Some of the transmission spectra are shown in Figure 4. For a fixed gap width of 10 nm, we deposited 0, 10, and 20 nm of additional alumina on top of the gap compared terahertz transmission through the gap and that through the gap with water layer on top. As shown in Figure 4A, increasing gap-water distances from 0 to 20 nm leads to a smaller transmittance decrease by the water layer on top, i.e., reduction in absorption, as field enhancement is larger near the gap. For a same gap-water spacing of 20 nm, however, water layer on top of 20 nm gap incorporates larger absorption, despite smaller field enhancement of the 20 nm gap compared to that of 5 or 10 nm gap (Figure 4B). This implies that field distribution as well as field amplitude should both be taken into consideration when understanding the absorption behavior.

To understand the absorption by the fringe fields, we consider (1) the relationship between absorption enhancement and local fields, and (2) distribution of electromagnetic fields above the nanogap. It has previously been shown that the former can be understood in context of the Fermi’s golden rule, assuming that terahertz absorption is essentially a dipole transition within an absorbing medium [15]. For molecules inside metallic nanogaps with high field enhancements the absorption cross section is given as:

$$\alpha =\frac{2\pi }{\hbar }\mu^{2}E^{2}\rho \left(\hbar \omega_{0}\right)N\frac{\hbar \omega_{0}}{S}=\frac{2\pi }{\hbar }\mu^{2}\rho \left(\hbar \omega_{0}\right)N\hbar \omega_{0}\frac{E^{2}}{S}$$

where *μ* is transition dipole of the absorbing system, *N* is number density of the molecules, *ρ* is density of states, and *E* and *S* are time averaged electric field and Poynting vector, respectively. The electromagnetic enhancement factor Σ therefore follows a ratio of near field enhancement factors *E*_enh_^2^/*S*_enh_, which can become as large as several thousands in slot antennas where electric field is much stronger than the magnetic field. This relation holds even when the electric field is nonuniform in space if there is no other path for energy loss other than the absorption by the molecules.

**Figure 5:**
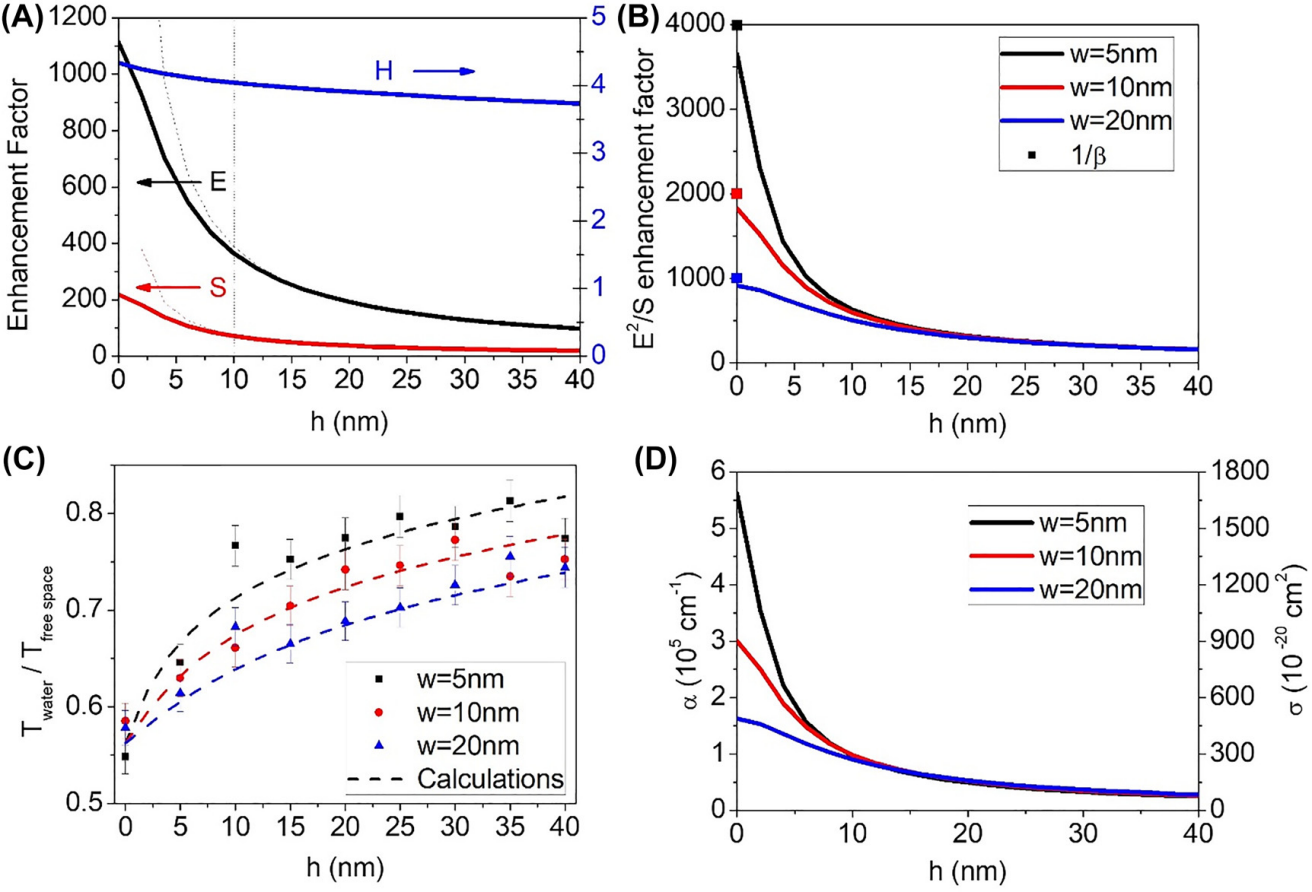
Absorption enhancement on top of nanotrenches at various distances. (A) Electromagnetic field distribution above a 10 nm gap obtained from modal expansion calculation. Dashed curves denote a 1/*h* fitting curve, fitting well in radiative zone (*h* > *w*) but deviating from the calculations in the reactive zone (*h* < *w*). The dashed vertical line denotes the boundary of radiative zone and reactive zone for the 10 nm gap. (B) Calculated absorption coefficient enhancement above 5, 10, and 20 nm gap samples. Inverse of gap coverage ratio is plotted as a dot in *y*-axis for comparison. (C) Plots of experimental (dots) and theoretical (dashed lines) transmission ratios for various gap sizes and gap-water distances. Normalized intensity transmission for nanogap with water is divided with that of nanogap without water. The error bars correspond to uncertainties introduced during repeated uses of the cover layer (see Supporting Information for details). (D) Effective absorption coefficient (left axis) and absorption cross section (right axis) of liquid water, corresponding to the transmission decrease shown in Figure 4C.

Spatial distribution of the fringe fields, determined from a modal expansion calculation, is described in Figure 5A, for the case of the 10 nm gap sample. Absorption enhancement factors for 5, 10, and 20 nm gaps are shown in Figure 5B. The figures imply that the near field can be described in terms of two distinct regimes: radiative zone (*w* < *h* < *d*, *d*: periodicity of the nanogap), and reactive zone (0 < *h* < *w*), following the familiar notations in antenna theory. In the radiative zone, the gap can be considered as a line radiation source, meaning that the scattering is cylindrical and Poynting vector should be proportional to 1/*r* for energy conservation. Meanwhile, the electric field also falls off as 1/*r* since the magnetic field outside the nanogap is not enhanced much and is nearly a constant. Therefore, the radiative zone possesses an absorption enhancement factor proportional to *E* ^2^/*S* ∼ 1/*r*, decaying significantly slower than in other commonly seen geometries such as a point-like gap. This implies that considerable amount of absorption can take place even at distances several times larger than the gap width. Note that ordinary cylindrical wave sources cannot incorporate such absorption enhancement, as they have no imbalance between electric and magnetic field amplitudes.

As the gap-water distance becomes smaller than the gap width (reactive zone), scattered fields from nanotrenches cannot be considered as coming from a line source anymore, thereby deviating from the 1/*r* curve. Instead, the fringe field shows complex polynomial dependence on *r*, which converges to zeroth order on the gap exit (*h* = 0). It should also be noted that the absorption enhancement factor at the gap is nearly equivalent to *β* = *A*_gap_/*A*_metal_. This can be understood in the context of energy conservation and diffraction theory. Since the far-field transmitted energy is solely from the energy flux at the gap, *S*_far_ = *S*_near_×(*A*_near_/*A*_far_). This leads to a relation for the near field Poynting vector enhancement *S*_enh_ = *t* ^2^/*β*. Also, the Kirchhoff integral formalism relates near field enhancement and far field transmittance via *E*_enh_ = *t*/*β*. This explains the observed dimensionless absorption enhancement factor by *E*_enh_^2^/*S*_enh_ = (*t*/*β*)^2^/(*t* ^2^/*β*) = 1/*β*, which is also consistent with analytical calculations.

Figure 5C shows the complete set of relative transmission versus gap-water distance for 5, 10 and 20 nm gap samples, where peak transmission of a nanogap with water on top is divided with that of nanogap without water. Considering that relative transmission increase by ALD deposition represents the amount of absorption from water layer replaced by the ALD-deposited alumina, one can estimate the effective absorption coefficient of water layer at certain position from following expression based on the Beer–Lambert law:

$$\frac{T\left(r_{2}\right)}{T\left(r_{1}\right)}=\text{exp}\left(-\overset{r2}{\underset{r1}{\int }}\alpha \left(r\right)\text{d}r\right)≃\text{exp}\left(-\alpha_{\text{avg}}\cdot \Delta r\right)$$

where *T*(*r*) is far field transmission when thickness of the ALD-deposited alumina is *r*, and *α*(*r*) is the effective absorption coefficient at location *r*, and Δ*r* = *r*_2_ − *r*_1_. Therefore, the 10% change in transmission for 5 nm gap-water distance on 5 nm gap implies that the 5 nm-thick water layer works as an absorbing layer with an average absorption coefficient of *α*_avg_ = −Δ*r* ^−1^ln(*T*(*r*_2_)/*T*(*r*_1_)) = −(5 nm)^−1^ln(0.1) = 4.61 × 10^5^ cm^−1^. As absorption coefficient of water at terahertz frequencies is around 150 cm^−1^, this is ∼3000-fold enhancement in absorption coefficient and accordingly, in absorption cross section (Figure 5D). It is also noticeable that non-negligible amount of absorption occurs at distances generally thought of as ‘outside the hot spot’, that is, several times of gap width away from the gap (nearing 300 at 15 nm away from the gap), arising from the relatively slow decay of electromagnetic field outside the high aspect ratio nanotrenches.

We expect that the beyond-hot-spot operation of nanotrenches should be especially useful for nanophotonic sensing of materials that are not compatible with conventional metal-target-metal gap geometry, as they can provide decent enhancement in light-matter interaction even when the target material can only lie on top of the gap. Such situation is expected to occur frequently in studies on biosystems, which often require a hydrophilic coating over the metallic nanostructures [32, 33]. Under such circumstances, the target biosystems needs to stay ∼15 nm away from the metallic surfaces and cannot benefit much from plasmonic field enhancement. Meanwhile, the nanotrenches provide ∼300-fold absorption enhancement even at such distances and, for instance, can induce terahertz absorption of 0.3 percent on a 5 nm-thick phospholipid bilayer with absorption coefficient of only 20 cm^−1^ [34]. Therefore, assuming a typical signal-to-noise ratio of >1000:1 for terahertz time-domain spectroscopy, the nanotrenches potentially enable single entity-level study of biosystems at terahertz frequencies. Also, as terahertz frequency range encompasses many intra- and inter-molecular vibrational modes of hydration water [35], DNA molecules [36], and hormones [37], etc., our work is expected to greatly benefit the field of nano-bio-plasmonics.

In summary, terahertz absorption of water layer on top of sub-20 nm-wide nanotrenches is investigated. Precise control over the gap-water distance confirms that electromagnetic absorption enhancement follows *E*_enh_^2^/*S*_enh_, even for spatially rapid-varying fields outside the gap. This explains the absorption behavior in both reactive and radiative zones of the nanogap, where the enhancement factor starts from inverse of gap coverage ratio and falls as 1/*r* for large distances. The relatively slow decay of absorption enhancement factor leads to considerable amount of absorption even at distances several times larger than the gap width, calling for an extended definition for ‘hot spot’. Also, as the cylindrical scattering can occur whenever the gap width is extremely sub-wavelength and the length is comparable to or larger than the wavelength, our scheme can be applied to extended gap structure operating at practically any wavelength range including near-, mid-infrared, and even microwaves [6, 38, 39]. Along with the experimental accuracy provided by the well-understood field profile, the nanotrench structure can pave way through various nanogap-assisted detection schemes that were previously inaccessible.

## Supporting Information Available

Detailed information on Kirchhoff integral formalism and modal expansion calculation, repeatability of the PDMS-based cover.

## Supplementary Material

Supplementary Material Details
