# Supplementary material for: Beyond-hot-spot absorption enhancement on top of terahertz nanotrenches
Source: Nanophotonics. 2022 May 25;11(13):3159–67. doi: 10.1515/nanoph-2022-0214 (PMC11501868; doi:10.1515/nanoph-2022-0214)
Supplement: Supplementary file 1 — Supplementary Material Details [file j_nanoph-2022-0214_suppl.docx]

**<Supporting Information>**

**Beyond-hot-spot absorption enhancement on top of terahertz nanotrenches**

Jeeyoon Jeong^1, †^, Dai-Sik Kim^2,3^, and Hyeong-Ryeol Park^3,^ **^‡^**

^1^Department of Physics and Institute of Quantum Convergence Technology, Kangwon National University, Gangwon 24341, Republic of Korea

^2^Department of Physics and Astronomy, Seoul National University, Seoul 08826, Republic of Korea

^3^Department of Physics, Ulsan National Institute of Science and Technology (UNIST), Ulsan 44919, Republic of Korea

Emails: ^†^peterjjy@kangwon.ac.kr, **^‡^**nano@unist.ac.kr

Keywords: Terahertz, nanogaps, absorption, hot spots, field enhancement

**S1. Estimation of near field enhancement at the gap exit with Kirchhoff integral formalism**

We follow the formalism introduced in Ref. 14 to relate the near field enhancement at the gap to the far field transmittance. When observation distance from the sample R is much larger than the wavelength λ, the far field electric field can be approximated as:

$$E_{far}=\left( \frac{e^{ikR}}{i\lambda R} \right)\int E_{near}dA_{near}=\left( \frac{e^{ikR}}{i\lambda R} \right)E_{near}A_{near}$$

where $A_{near}$ is the area on the sample where the electric field is distributed. For nanogaps used in the experiment $A_{near}=A_{gap}$ since the transmitted waves exclusively come from the gap. Since normalization is made with respect to transmittance of bare substrate (which will be denoted as S in super- or subscript), normalized transmittance t relates with the near field enhancement factor $E_{near}^{gap}/E_{near}^{S}$ as:

$$t=\left( \frac{E_{far}^{gap}}{E_{far}^{S}} \right)=\frac{E_{near}^{gap}}{E_{near}^{S}}\times\frac{A_{gap}}{A_{S}}, \frac{E_{near}^{gap}}{E_{near}^{S}}=\frac{t}{\beta}$$

with the coverage ratio $\beta=A_{gap}/A_{S}$.

**S2. Modal expansion calculation for sub-20 nm-wide, ~100 μm-long nanogap structures**


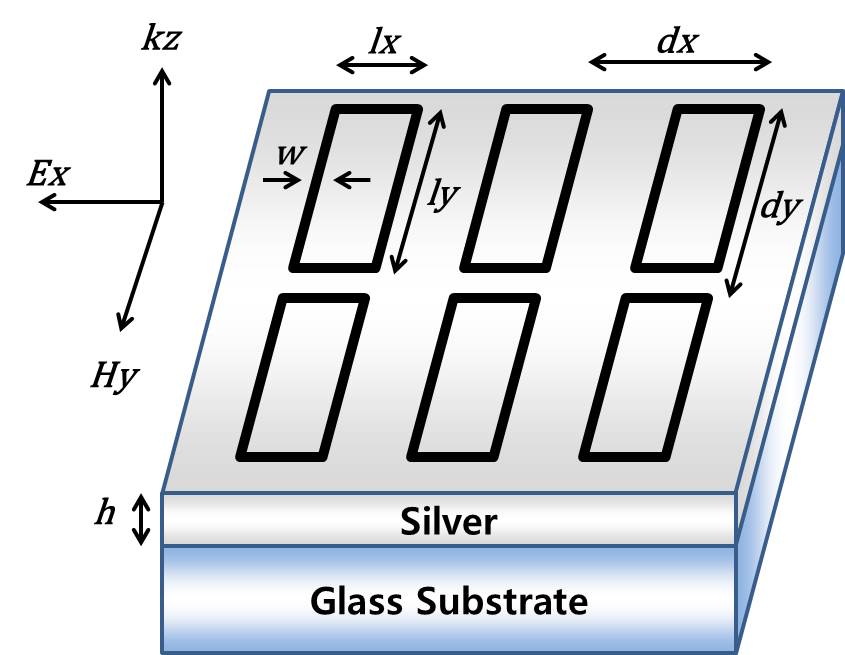


Figure S1. Geometry of the nanogap sample and incident electromagnetic field used in the experiment and calculation.

An analytical calculation based on modal expansion method is used to calculate the field profile near the gap, since simulation methods such as finite-difference time domain (FDTD) method or finite element method (FEM) is not capable of handling nanometer-size grids under wavelengths of several hundred microns. Also, analytic solutions can provide insights into physical origins of the observed field profile. Modal expansion calculation determines the coupling of nanogap waveguide mode to the incoming and outgoing electromagnetic waves to characterize the transmission. For the nanogap array used in the experiment (Figure S1), following the convention described in Ref. 29-30 of the main text, the electric field at the gap can be expressed as:

$$E_{gap}=\frac{G_{V}I_{0}}{\left( G_{I}-\Sigma\right)\left( G_{III}-\Sigma\right)-G_{V}^{2}}, G_{V}=\frac{1}{k_{0}h\times sinc\left( \beta h \right)}, \beta=\sqrt{\epsilon_{d}k_{0}^{2}-\left( \frac{\pi}{l} \right)^{2}} (l=l_{x}+l_{y})$$

While the metal was considered as perfect electric conductor in previous studies, however, gap plasmon effect needs to be considered for small gap sizes to account for wavevectors leaking into the metals with finite permittivity $\epsilon_{m}$ (Ref. 30, 31 of the main text). We incorporate the effective index method into this formalism to correctly account for the local increase of refractive index inside the gap by considering the leaking wavevector $k_{m}$ as follows.

$$G_{V}=\frac{1}{k_{0}h\left( 1+\frac{k_{m}^{2}}{\epsilon_{d}k_{0}^{2}} \right)\times sinc\left( \beta h \right)}, \beta=\sqrt{\epsilon_{d}k_{0}^{2}+k_{m}^{2}-\left( \frac{\pi}{l} \right)^{2}}$$

$$k_{m}^{2}= -\frac{2\left( 1+\sqrt{1-k_{0}^{2}\left( \frac{w\epsilon_{m}}{\epsilon_{d}} \right)^{2}*\left( \epsilon_{m}-\epsilon_{d} \right)} \right)}{\left( \frac{w\epsilon_{m}}{\epsilon_{d}} \right)^{2}}$$

The electric and magnetic field outside the gap is then expressed as:

$$E_{x}(\vec{r})=E_{gap}\times\sum_{k_{x}, k_{y}} \frac{J\left( k_{x}, k_{y} \right)}{dxdy}\exp\left( i\vec{k}\cdot\vec{r} \right), H_{y}(\vec{r})=\frac{E_{gap}}{Z_{0}}\times\sum_{k_{x}, k_{y}} \frac{J\left( k_{x}, k_{y} \right)}{dxdy}\frac{k_{x}^{2}+k_{z}^{2}}{k_{0}k_{z}}\exp\left( i\vec{k}\cdot\vec{r} \right)$$

$$J=sinc\left( k_{x}w/2 \right)\left\{ sinc\left( \frac{\pi+k_{y}l}{2} \right)+sinc \left( \frac{\pi-k_{y}l}{2} \right) \right\}$$

where the summation is over integer multiples of ${2\pi}/{dx}$ and ${2\pi}/{dy}$ for $k_{x}$ and $k_{y}$, respectively. As the sinc function in J converges quickly as a function of $k_{x}$, field profile in and near the gap can be calculated accurately with relatively low number of included modes.

**S3. Repeatability of the PDMS-based cover**


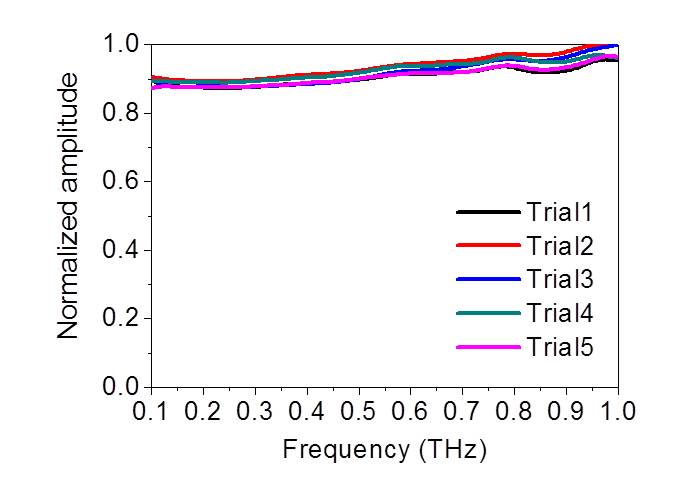


Figure S2. Repeated transmission measurement on water layer between the substrate and the cover. In each trial, the cover is detached and re-attached to the sample and forms a new water reservoir every time. The transmitted signals are normalized with respect to the same structure without water layer.

PDMS is chosen as a spacer material because it meets the following requirements: (1) the thickness can be controlled by adjusting the spin coating parameters, (2) its affinity to glass assures complete sealing of water on top of nanogaps, and (3) the fabricated cover can be reused many times since the adhesion to the substrate is strong but not permanent. The third requirement is especially important in this experiment because the sample needs to go through additional atomic layer deposition (ALD) after each measurement. But it is hard to meet the requirements (2) and (3) at the same time since soft, reusable materials such as double-sided tapes usually suffer from deformation during repeated measurements. Cover with spin-coated PDMS spacer shows excellent performance in reusability as shown in Figure S2, where the transmittance of the substrate/water/cover multilayer is 0.89 and is nearly unchanged between each trial ($\pm0.01$). This is the largest source of error in this experiment and is represented as error bars in Figure 4(c).
